# Dependence of the Optical Constant Parameters of p-Toluene Sulfonic Acid-Doped Polyaniline and Its Composites on Dispersion Solvents

**DOI:** 10.3390/molecules25194414

**Published:** 2020-09-25

**Authors:** Fahad Usman, John Ojur Dennis, Fabrice Meriaudeau, Abdelaziz Yousif Ahmed, Khe Cheng Seong, Yap Wing Fen, Amir Reza Sadrolhosseini, Bashir Abubakar Abdulkadir, Ridwan Tobi Ayinla, Wan Mohd Ebtisyam Mustaqim Mohd Daniyal, Nur Alia Sheh Omar, Nissren Tamam, Abdelmoneim Sulieman

**Affiliations:** 1 Department of Fundamental and Applied Sciences, Universiti Teknologi PETRONAS, Malaysia, Seri Iskandar, Perak 32610, Malaysia; johndennis@utp.edu.my (J.O.D.); chengseong.khe@utp.edu.my (K.C.S.); abubakarbashir150@gmail.com (B.A.A.); 2 Department of Physics, Al-Qalam University, Katsina, Katsina, PMB 2137 Katsina, Nigeria; 3 ImViA EA 7535, Team IFTIM, Université de Bourgogne, 21000 Dijon, France; Fabrice.Meriaudeau@u-bourgogne.fr; 4 Department of Electrical and Electronic Engineering, Universiti Teknologi PETRONAS, Seri Iskandar, Perak 32610, Malaysia; azez23101@gmail.com; 5 Department of Physics, Universiti Putra Malaysia, Serdang, Selangor 43400, Malaysia; yapwingfen@upm.edu.my; 6 Institute of Advanced Technology, Universiti Putra Malaysia, Serdang, Selangor 43400, Malaysia; amir1348@gmail.com (A.R.S.); wanmdsyam@gmail.com (W.M.E.M.M.D.); nuralia.upm@gmail.com (N.A.S.O.); 7 Department of Nanoscience and Nanotechnology, Université Grenoble Alpes, 621 Avenue Centrale, 38400 Saint-Martin-d’Hères, France; rtayinla@student.lautech.edu.ng; 8 Physics Department, College of Sciences, Princess Nourah Bint Abdulrahman University, P.O. Box 84428, Riyadh 11671, Saudi Arabia; nissrentamam@gmail.com; 9 Radiology and Medical Imaging Department, College of Applied Medical Sciences Prince Sattam bin Abdulaziz University, P.O. Box 422, Alkharj 11942, Saudi Arabia; a.sulieman@psau.edu.sa

**Keywords:** p-toluene sulfonic acid-doped polyaniline, optical constant parameters, chitosan, reduced graphene-oxide, surface plasmon resonance (SPR)

## Abstract

The optical constants of Para-Toluene sulfonic acid-doped polyaniline (PANI), PANIchitosan composites, PANI-reduced graphene-oxide composites and a ternary composite comprising of PANI, chitosan and reduced graphene-oxide dispersed in diluted p-toluene sulfonic acid (PTSA) solution and *N*-Methyl-2-Pyrrolidone (NMP) solvent have been evaluated and compared. The optical constant values were extracted from the absorbance spectra of thin layers of the respective samples. The potential utilization of the materials as the active sensing materials of surface plasmon resonance biosensors has also been assessed in terms of the estimated value of the penetration depth through a dielectric medium. The results show a reasonable dependence of the optical constant parameters on the solvent type. Higher real part refractive index (*n*) and real part complex dielectric permittivity (*ε*’) values were observed for the samples prepared using PTSA solution, while higher optical conductivity values were observed for the NMP-based samples due to their relatively higher imaginary part refractive index (*k*) and imaginary part complex dielectric permittivity (*ε*″) values. In addition, NMP-based samples show improvement in terms of the penetration depth through a dielectric medium by around 9.5, 1.6, 4.4 and 2.9 times compared to PTSA-based samples for the PANI, PANI-chitosan, PANI-RGO and the ternary composites, respectively. Based on these, it is concluded that preparation of these materials using different dispersion solvents could produce materials of different optical properties. Thus, the variation of the dispersion solvent will allow the flexible utilization of the PANI and the composites for diverse applications.

## 1. Introduction

Conducting polyaniline, especially in its doped form (emeraldine salt or ES), features many potential industrial applications due to its unique optical, electrical and optoelectronic properties. However, ES is considered a poorly processible material due to its increased crystallinity or crystalline structure, which affects its dispersibility [1]. This hinders many of its industrial applications. Fortunately, it was found that ES can be processed using some selected functional protonic acids and selected organic solvents [2,3]. This opens the way for many important findings on the processibility of doped polyaniline. For example, the electrical, optical, crystalline and dielectric properties of the resulting processible ES have been investigated comprehensively [4]. Moreover, the optical properties of the ES were found to differ with different solvents. For example, camphor sulfonic acid-doped polyaniline dispersed in meta cresol was found to possess a region of carrier tails in place of the well-known polaronic peaks that were observed in the doped polyaniline dispersed in chloroform [2,5].

It is important to assess the optical constant parameters, such as the refractive index, the dielectric constants and the optical conductivity of materials, before their applications are widely considered [6,7]. This is due to the diverse optical property requirements for various applications. For example, low refractive index materials are ideal for the fabrication of optical components such as reflectors, filters, band-passes and photonic crystals. This is in addition to the fabrication of devices such as lasers, LEDs, and solar cells [8]. On the other hand, high refractive index polymeric materials are reported to be required for anti-refractive coatings, micro-lenses for CMOS image sensors, encapsulants for LEDs, and high-n thermoplastic lens applications [9]. It could be concluded that the processability of doped polyaniline and the investigation of its electrical, optical, crystalline and dielectric properties are well developed [1,4,10]. However, investigation related to the effect of dispersion solvent on its optical constant parameters is lacking.

As such, this work is aimed at comparing the optical constant parameters of p-toluene sulfonic acid (PTSA) doped polyaniline (PANI), PANI-chitosan, PANI-reduced graphene oxide (RGO) and the ternary composites of PANI, RGO and chitosan that are prepared using two different dispersion solvents; diluted PTSA solution and *N*-Methyl-2-Pyrrolidone (NMP). In addition, the applicability of the materials (samples) in surface plasmon resonance (SPR) sensing application is also explored. This is an extension of our previous work on the optical constant of p-toluene sulfonic acid-doped polyaniline processed in PTSA solution [7]. It is observed that the dispersion solvent variation can greatly affect the optical constant values of the PANI and the composites (samples). Therefore, the solvent dependence variability of the optical constants of these samples could allow the flexible utilization of the PANI and the composites for various purposes, such as energy, environmental and sensing applications.

## 2. Materials and Methods

### 2.1. Materials

Toluene-4-sulfonic acid monohydrate (PTSA)-Merck, *N*-Methyl-2-Pyrrolidone (NMP), chitosan-Sigma Aldrich (medium molecular weight (190-310 kDa) with 75–85% degree of deacetylation) were all supplied by Avantis chemicals supply (Ipoh-Perak, Malaysia). All the chemicals used were of analytical grade. PANI, PANI-chitosan, PANI-RGO, and the ternary composites of PANI, RGO and chitosan, were all synthesized at our laboratory.

### 2.2. Synthesis and Characterization

The methods for the synthesis and characterization of PANI, PANI-chitosan, PANI-RGO and the ternary composites were explained comprehensively in our previous work [7]. In addition to that, the UV-Visible characterization for the NMP-processed PANI and the composites was conducted for comparison with the PTSA-based counterpart. The surface morphology of the thin films of these materials was studied using Field Emission Scanning Electron Microscopy (FESEM) imagery, obtained using VPFESEM, Zeiss Supra55 VP (Oberkochen, Germany). The potential of the materials for surface plasmon resonance sensing application has been investigated through the estimation of the penetration depth of plasmon waves through a dielectric medium [11,12].

### 2.3. Preparations of Thin Layer

The thin layer preparations for the PTSA-based PANI and the composites were explained previously [7]. For the NMP-based materials, 15 mg/mL solution (dispersion) of the materials in NMP was formed and utilized. The dispersion process was followed by a constant magnetic stirring for 12 h at 600 rpm in each case. The thin layers were deposited on microscopic glass substrates (Menzel™ Microscope Coverslips) using a POLOS™ spin coater set at 1500 rpm for 1 min. The glass substrates were washed with acetone and deionized water prior to the deposition in order to eliminate dust and impurities. The deposited layers were then kept in an oven for 4 h at 40 °C before storing them in an undisturbed place for further characterizations. The thicknesses of the respective thin film layers were estimated by surface roughness tester (SV-mutitoyo-3000) using the scratch after deposition method.

## 3. Results

### 3.1. Comparison of UV-Vis Absorption Spectra

**Figure 1:**
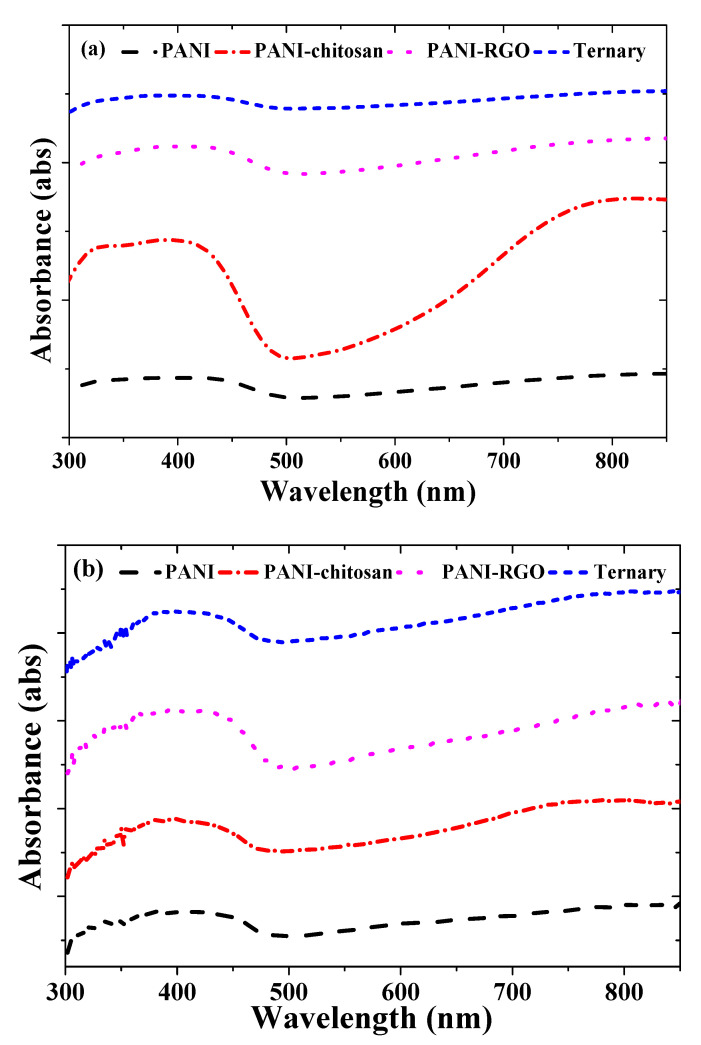
UV-VIS spectra of (**a**) PANI, PANI-chitosan, PANI-RGO and Ternary composites dispersed in PTSA (**b**) PANI, PANI-chitosan, PANI-RGO and Ternary composites dispersed in NMP.

The UV-VIS absorption spectra for PANI, PANI-chitosan, PANI-RGO and the ternary composites are shown in Figure 1a,b for the PTSA and NMP-based solvents, respectively. Previously, three characteristic peaks attributable to the π–π* transition of the benzenoid rings, localized polarons and delocalized polarons have been reported at around 325, 433 and 800 nm, respectively, for the PTSA-based materials [7]. These peaks are more obvious in the case of the PANI-chitosan composite as shown in Figure 1a. However, the absorption spectra presented in Figure 1b for the NMP-based materials are closer to what was observed for the CSA-doped polyaniline dispersed in meta cresol solvent [2,5]. As such, in Figure 1b, the delocalized polaron band is obviously replaced by a free carrier tail.

The manifestation of the free carrier has been attributed to the greater delocalization of the polaron band in the expanded coil-like conformation of doped polyaniline due to the removal of twist defects between aromatic rings [13,14]. In addition, the weakening or even disappearance of the band attributable to the π–π* transition of the benzenoid rings has been attributed to the elimination of the energy gap between the *π* band and the polaron band [2,13,14]. As reported previously, a similar overlapped or weakening feature has been observed at the region of π–π* transition and the localized polarons [7,15]. The same effect is observed for the NMP-based materials as shown in Figure 1b. In general, these features prove the presence of p-toluene sulfonic acid-doped polyaniline in all of the composites. In addition, the modification of the PANI spectra compared to that of the composites further confirms the formation of the composites [7].

### 3.2. Field Emission Scanning Electron Microscopy (FESEM)

**Figure 2:**
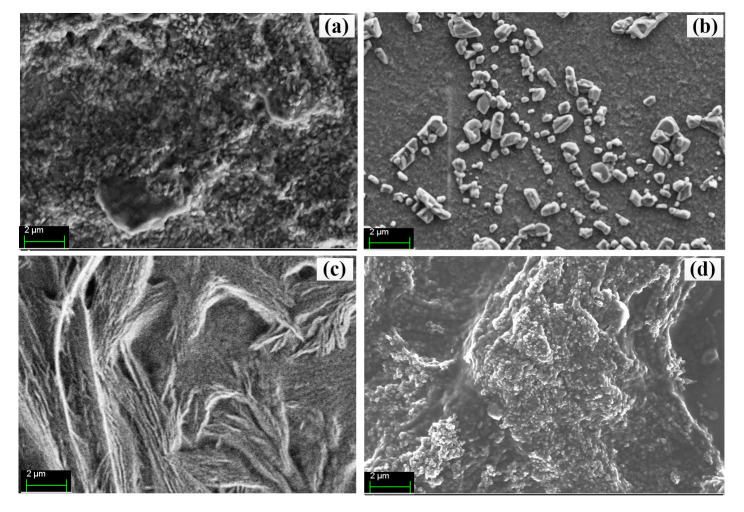
FESEM images of (**a**) PANI, (**b**) PANI/chitosan, (**c**) PANI/RGO and (**d**) Ternary composites processed using NMP.

The surface morphology of the PANI, PANI-chitosan, PANI-RGO and the ternary composite films processed using PTSA solution has been studied using FESEM, and presented in our previous work [7]. In summary, PANI was found to feature a long fibrillar chain of short granular structures, while PANI-chitosan depicted a flat flags surface with dispersed granular structures due to PANI. In addition, a fibrilla–lamellar structure was observed in the case of PANI-RGO. Furthermore, a nanoporous-like structure attributable to the uniform dispersion of RGO in chitosan was observed for the ternary composite. Figure 2a–d shows the FESEM images for the PANI, PANI-chitosan, PANI-RGO and the ternary composite films processed using NMP, respectively. Interestingly, structures similar to that of the PTSA-based samples are observed. However, few changes are observed. For example, reduction in the sizes of the particles for the PANI, PANI-chitosan and the ternary composites is noted. This is due to the utilization of NMP in place of the sulfonated acid (PTSA), which has the potential of clumping PANI particles at the risk of low conductivity, especially when the concentration is ≥0.4 M [16]. In addition, clearer and improved distribution of particles attributable to the presence of PANI is observed in Figure 2d. This is due to the presence of chitosan, which is known to improve the processibility of PANI [15,17,18]. More importantly, the roughness and the pore spaces of the ternary composite film could result in better absorption of the analyte in sensing application [19].

### 3.3. Comparison of Complex Refractive Index Parameters

**Figure 3:**
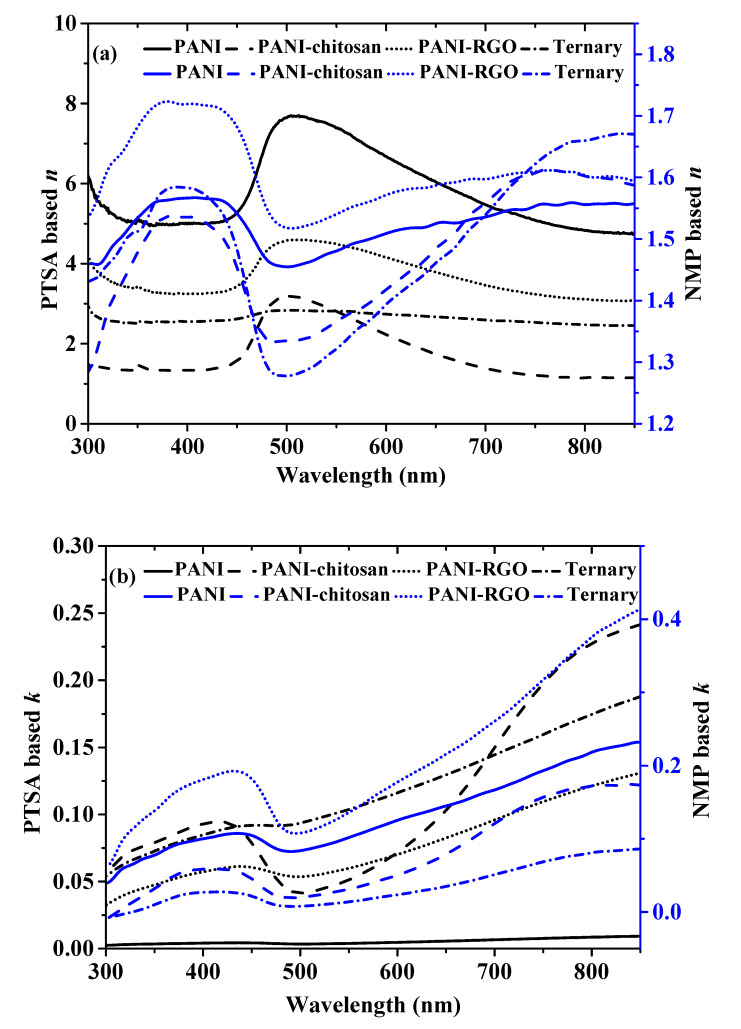
(**a**) Real part refractive index of PANI, PANI-chitosan, PANI-RGO and Ternary composites dispersed in both PTSA and NMP and (**b**) Imaginary part refractive index of PANI, PANI-chitosan, PANI-RGO and Ternary composites dispersed in both PTSA and NMP.

In order to investigate how the propagation of optical radiation through the PANI, PANI-chitosan, PANI-RGO and the ternary composites differ with PTSA and NMP solvents, their real part refractive index (*n*) values are compared, as shown in Figure 3a. The formula (Equation (1)) and the method for the evaluation of *n* were reported previously [7], where R is reflectance and k is the extinction coefficient (imaginary refractive index) given by *k* = ((2.303 *A*/*d*)*λ*/4π). Moreover, A represents absorbance, λ represents the wavelength of the light waves and *d* represents the thickness of the thin layer of the materials. Surprisingly, the obtained *n* values for the NMP-based materials are quite less than the previous value observed for the PTSA-based materials as depicted in Figure 3a [7]. It could be observed that the values are closer to many available reports on the refractive index of doped polyaniline [20,21]. This implies that the PTSA solution is the main contributor of the high refractive index observed in the materials dispersed in PTSA solution, as shown in Figure 3a [7]. For example, the peak *n* value of PANI dispersed in PTSA is around 7.7 at 505 nm, while the peak *n* value of 1.6 at 431 nm was observed for the PANI dispersed in NMP. The same trend is also observed in the other materials. This indicates that the materials dispersed in PTSA are denser than the NMP-based ones due to their greater proportion of sulfur, which is known to raise refractive index values due to its high value of molecular refraction [7,9]. The higher *n* value of the materials dispersed in the PTSA compared to the NMP-based materials could also be explained in terms of the hydrogen bond formation between the PTSA and the polyaniline functional group [22]. It could also be noticed that the lowest *n* values for the materials dispersed in both the PTSA and the NMP are found in the PANI-chitosan and the ternary composite, which may be due to the low density of chitosan [23]. Moreover, the change in the *n* values for the different materials proves the existence of some interaction between photons and electrons in the thin layers of the materials in different ways [6]. This, by extension, confirms the presence of materials for different applications. Therefore, the tunable and different dispersive nature of both the materials dispersed in PTSA and NMP could be exploited for different industrial applications.

(1) $$n=\left[\frac{1+R}{1-R}\right]+\sqrt{\frac{4R}{{\left(1-R\right)}^{2}}}-k^{2},$$

On the other hand, as shown in Figure 2b, the higher imaginary refractive index values of *k* are observed for the materials dispersed in PTSA compared to NMP-based materials, which is due to the higher scattering of photons by the PTSA-based materials relative to the NMP-based materials [6,7]. In addition, the lower values observed for the NMP-based materials indicate the more transparent nature of the materials relative to the PTSA-based counterpart.

### 3.4. Comparison of Complex Dielectric Constant

**Figure 4:**
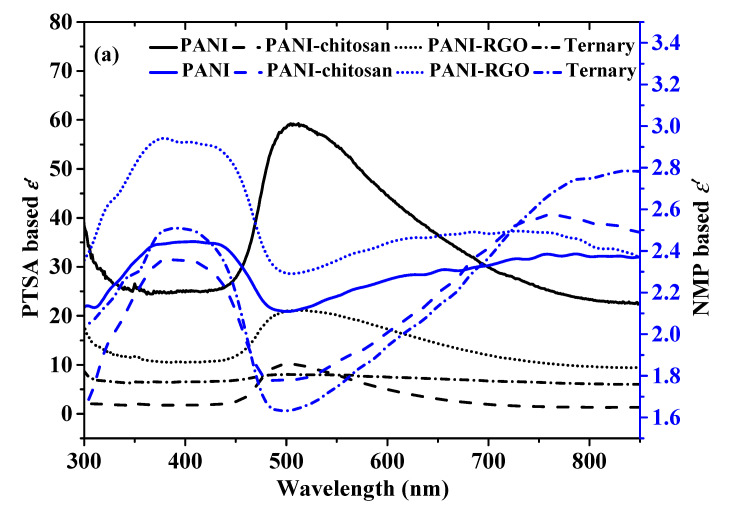

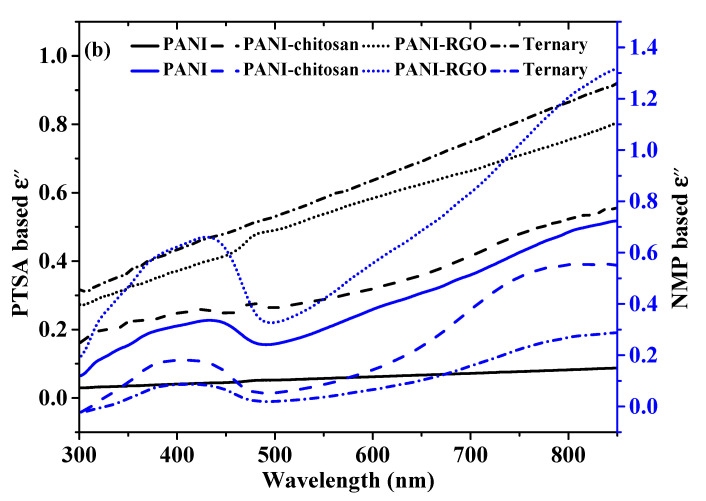
(**a**) Real part dielectric constant of PANI, PANI-chitosan, PANI-RGO and Ternary composites thin layers dispersed in PTSA and NMP (**b**) Imaginary dielectric constant of PANI, PANI-chitosan, PANI-RGO and Ternary composites thin layers dispersed in PTSA and NMP.

The complex dielectric constant is another important optical parameter that is composed of real and imaginary parts. The real part of the dielectric constant (*ε*’) indicates the ability of a material to store electric energy. It could also be described in terms of the ability to allow the passage of an electric field through a material [24]. On the other hand, the imaginary part (*ε*″) provides a measure of the dissipation of the electromagnetic wave in the film of a material [6]. The formula for the calculations of the components of this complex dielectric constant has been described previously (Equations (2) and (3)) [7]. The values of the real and the imaginary part of the dielectric constant based on the two dispersion solvents are shown in Figure 4a,b, respectively. It could be observed that the Figures for the dielectric constant values (Figure 4a,b) are closely related to that of the complex refractive index shown in Figure 3a,b. This proves the relationship between the two parameters [7,25]. The higher *ε*’ values in PANI and PANI-RGO for both the PTSA and NMP-based materials show the existence of more energy density of states, which results in an increased polarization [7,26,27]. For the same reason, PTSA-based materials also show higher *ε*’ values compared to NMP-based materials (Figure 3a).

(2) $$\varepsilon^{\prime }=n^{2}-k^{2},$$

(3) $$\varepsilon^{\prime \prime }=2nk,$$

Figure 3b shows that the values of *ε*″ for both the PTSA and NMP-based material increase with wavelength. It could also be observed that the dissipation of electrical energy varies with different dispersion solvents. For example, the ternary composite dispersed in PTSA shows the highest *ε*″ compared to the other materials. However, the lowest values of *ε*″ were found for the ternary composite layer dispersed in NMP. This indicates that the ternary thin layer dispersed in PTSA dissipates more electrical energy than the NMP-based films [6]. This further proves the tuning ability of doped polyaniline and its composite by using different dispersion solvents which can be exploited in different applications.

### 3.5. Comparison of Optical Conductivity

**Figure 5:**
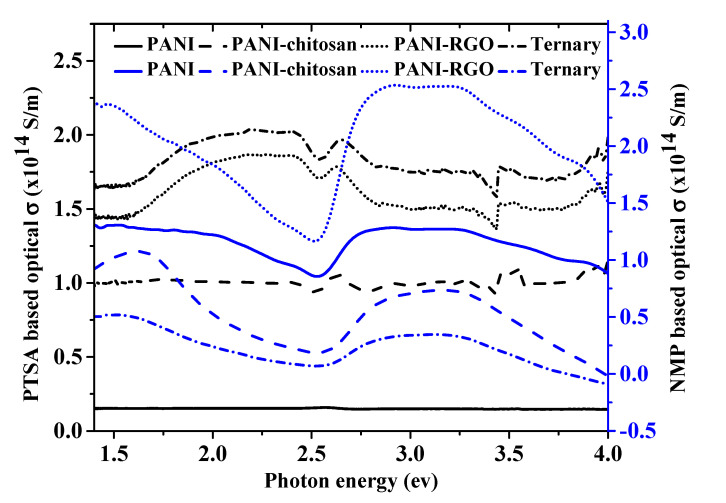
The optical conductivity values, *σ* versus incident photon energy for PANI, PANI-chitosan, PANI-RGO and Ternary composite.

The formula for the evaluation of the optical constant has also been described in our previous work (Equation (4)) [7]. Figure 5 compares the optical conductivities of the PANI and the composite materials dispersed in PTSA and NMP. The optical conductivity of PTSA-based materials was explained previously [7]. The relatively higher optical conductivity for the NMP-based materials could be attributed to their higher absorbance values as evidenced in their *k* and *ε*″ values, which are in direct proportion with the absorption co-efficient value [26]. The materials dispersed in PTSA are expected to have a higher optical conductivity due to their greater charge carrier concentration [26]. However, the higher optical conductivity of the NMP-based materials has proven the domination of the absorption effect over the charge carrier concentration.

(4) $$\sigma =\frac{\alpha nc}{4\pi },$$

where *σ* is the optical conductance, *c* is the velocity of the radiation in space, *n* is the refractive index and *α* is the absorption coefficient.

### 3.6. Applications of the Materials for Surface Plasmon Resonance Biosensors

Surface plasmon resonance (SPR) biosensors are among the most promising sensing devices due to their high sensitivity, real-time measurement, non-invasive measurement, label-free measurement and non-requirement of electrodes [28]. The design of these biosensors is guided by knowledge of the penetration depth of surface plasmon waves. The penetration depth is defined as the distance from the metal–dielectric interface at which the amplitude of the field becomes 1/*e* of the value at the interface [12,28]. It is classified as either the penetration depth through the dielectric medium ($\delta_{d})\;$ or the penetration depth through the metal medium ($\delta_{m})\;$. The penetration depth through the dielectric medium gives the measurement of the length over which the surface plasmon is sensitive to the changes in the refractive index of the dielectric medium [12]. A good SPR sensor is expected to possess a high ($\delta_{d})\;$ value [28]. The dielectric constant value of gold from the literature and that of the materials were substituted in Equation (5) in order to find the penetration depth through the composite materials ($\delta_{d})\;$ adjacent to the gold film [21].

(5) $$\delta_{d}=\frac{\lambda_{0}}{2\pi }{\left[\frac{\varepsilon_{m}^{\prime }+\varepsilon_{d}}{\left(\varepsilon_{d}\right)}\right]}^{1/2},$$

where λ_0_ is the free space wavelength, and $\varepsilon_{m}^{\prime }\;$ and ε*_d_* are the real part dielectric constants of gold and the material adjacent to gold, respectively.

**Figure 6:**
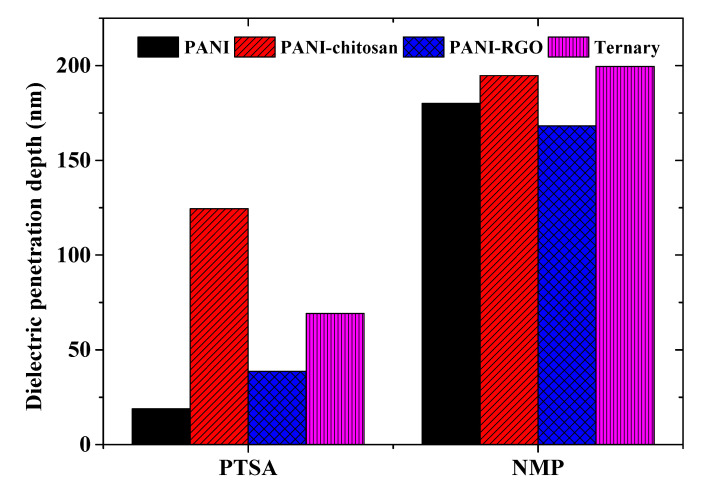
Penetration depths through a dielectric medium for PTSA and NMP-based samples.

The penetration depths for both the PTSA and NMP-based samples are shown in Figure 6 at 633 nm wavelength. It could be observed that the penetration through a dielectric medium for the NMP-based samples is about 9.5, 1.6, 4.4 and 2.9 times greater than the values for the PTSA-based samples for the PANI, PANI-chitosan, PANI-RGO and the ternary composites films, respectively. Therefore, the NMP-based materials are ideal SPR sensitive layers compared to the PTSA-based ones. However, higher dielectric constant values of the PTSA-based materials could be advantageous in other applications, such as in the development of supercapacitors [29,30,31].

## 4. Conclusions

The successful synthesis and characterization of the PANI, PANI-chitosan, PANI-RGO and the ternary composites was presented in our previous work. In this work, the optical constant parameters of these materials (samples) have been evaluated from the absorbance spectra of thin films of the respective samples. Diluted PTSA and NMP were used as the dispersion solvents for the materials in order to compare their optical constant values. The applicability of the PTSA and the NMP-based materials in SPR sensing application was also assessed in terms of the penetration depth through a dielectric medium. It is observed that the optical constant parameters differ with different dispersion solvents. The PTSA-based materials show higher *n* and *ε*’ values, while the NMP-based samples show higher optical conductivity values due to their higher absorbance value. In addition, the NMP-based samples also show improvement in terms of the penetration depth through a dielectric medium by around 9.5, 1.6, 4.4 and 2.9 times compared to PTSA-based samples for PANI, PANI-chitosan, PANI-RGO and the ternary composites, respectively. Hence, NMP-based samples are more promising in terms of SPR sensing application, while PTSA-based samples could be promising in other applications. The dispersive and the solvent dependence of the optical parameters of these materials could therefore be exploited for various applications according to their desired optical properties.
